# IMMEDIATE AND FOLLOW-UP EFFECTS OF A POSTURE EDUCATION PROGRAM FOR ELEMENTARY SCHOOL STUDENTS

**DOI:** 10.1590/1984-0462/;2017;35;2;00013

**Published:** 2017-05-15

**Authors:** Natália Brites dos Santos, Juliana Adami Sedrez, Cláudia Tarragô Candotti, Adriane Vieira

**Affiliations:** a Universidade Federal do Rio Grande do Sul (UFRGS), Porto Alegre, RS, Brasil.

**Keywords:** posture, health education, child health

## Abstract

**Objective:**

To assess the short- and medium-term effects of the posture education program (PEP) for students of elementary school regarding theoretical knowledge and posture during activities of daily living (ADLs).

**Methods:**

The sample consisted of 38 students (aged 8-12 years) in the third grade of elementary school in Porto Alegre, Rio Grande do Sul (Southern Brazil). The children were evaluated in three moments: prior to attending the PEP (pretest); after attending the PEP (post-test); and five months after the conclusion of the PEP, immediately after a learning review of four lessons (five months follow-up). The posture during ADLs and the theoretical knowledge about spine and body posture were assessed, based on specific instruments (layout for assessing the dynamic posture - LADy; and questionnaire). The Friedman test, *post hoc* Wilcoxon test, and Bonferroni correction were applied to identify the differences among the evaluative moments, as they are statistically significant at α<0.05.

**Results:**

No statistically significant difference was found between the post-test and follow-up concerning the theoretical knowledge. In addition, no statistically significant difference was found between post-test and follow-up in relation to ADLs; however, the performance of students was higher in the post-test and follow-up, when compared with the pretest.

**Conclusions:**

Immediately after the PEP’s conclusion, the students improved their posture in ADLs. These positive effects and the theoretical knowledge were retained in the follow-up (after the review lessons).

## RESUMO

**Objetivo:**

Avaliar os efeitos em curto e médio prazo de um Programa de Educação Postural (PEP) para escolares do ensino fundamental sobre o conhecimento teórico e o modo de execução das atividades de vida diária (AVDs).

**Métodos:**

Amostra composta de 38 escolares (8-12 anos) do terceiro ano do ensino fundamental de uma escola de Porto Alegre, Rio Grande do Sul. Os escolares foram avaliados em três momentos: antes de participar do PEP (pré-teste); após participação no PEP (pós-teste); e após cinco meses da participação no PEP, imediatamente após um reforço de aprendizagem de quatro aulas (*follow-up* cinco meses). A avaliação das AVDs e o nível de conhecimento teórico sobre a coluna vertebral e a postura corporal foram avaliados por instrumentos específicos (*layout for assessing the dynamic posture* - LADy; e questionário). Para verificar as diferenças entre os momentos avaliativos foram realizados o teste de Friedman e *post hoc* o teste de Wilcoxon e correção de Bonferroni (α<0,05).

**Resultados:**

Quanto ao conhecimento teórico, não houve diferença significativa entre pós-teste e *follow-up*. Na avaliação das AVDs, o desempenho dos escolares foi superior no pós-teste e *follow-up* quando comparados com o pré-teste, e na comparação entre os escores pós-teste e *follow-up* não houve diferença significativa.

**Conclusões:**

Constatou-se que o PEP apresentou efeito positivo imediatamente após o seu término na execução das AVDs e que houve manutenção desses efeitos e do nível de conhecimento teórico no *follow-up* após o reforço de aprendizagem.

## INTRODUCTION

Spinal pain and postural problems are quite prevalent in modern society. Classified among the most common health problems worldwide, these problems often prevent individuals from carrying out the activities of daily living (ADLs), and limit the active life of workers.^1^ In addition, epidemiological and clinical research data have been indicating high prevalence of back pain and postural deviations, among children and adolescents, which show that intervention actions are necessary for these groups.^2^ ^,^ ^3^ ^,^ ^4^ ^,^ ^5^

Studies highlight the multifactorial etiology for back pain and postural problems.^4^ ^,^ ^6^ Among various risk factors identified in the literature, it is possible to observe risk factors that associate pain and postural problems among young people with inadequate school furniture,^4^ ^,^ ^6^ ^,^ ^7^ and with body posture adopted in certain situations, such as when carrying school supplies,^4^ sitting,^5^ ^,^ ^8^ and sleeping.^5^ The body posture adopted during ADLs, which is considered a risk factor, determines the amount and distribution of effort over all segments of the body and may exacerbate or relieve the burden on the spine.^9^

Childhood is the most important period for the musculoskeletal development of an individual, during which postural alterations are more likely to be prevented and treated. Therefore, if prevention is initiated during early school years, young people may learn to adopt adequate movement patterns and will not need to fix inefficient and inadequate habits. Furthermore, it is possible to offer various learning review activities during school life, so that a large percentage of the population can be educated when prevention is carried out in school.^10^ ^,^ ^11^ ^,^ ^12^ In addition, proper posture in childhood and/or correction of postural deviations during this period leads to the maintenance of appropriate postural patterns in adulthood.^13^ Therefore, we can assume that the implementation of educational programs, also named back schools, could reduce or prevent the onset of postural problems in childhood.

Studies showed that back school participants tend to positively change their posture during ADLs, and improve their theoretical knowledge about the spine immediately after attending the posture education program (PEP).^14^ ^,^ ^15^ However, although there are positive immediate effects of back schools for schoolchildren, studies aimed at identifying whether these changes are permanent are still scarce. To this end, a study evaluated the effects of a PEP to children and adolescents eight months after the end of the program (follow-up)*,* and observed that the knowledge about the dynamic posture acquired during the intervention had not been retained after a period of eight months.^16^ In this context, the study showed that an interval of this magnitude between PEP’s conclusion and the follow-up, without guidance or learning review sessions may have negatively impacted the program’s results on the long term. These indicate the relevance of an intervention that proposes a follow-up preceded by strategies for reviewing and strengthening learning, as proposed in this study.

Therefore, considering the findings in the literature indicating immediate positive effects of back schools for schoolchildren, and the lack of studies to verify the effect of back schools in medium term, this study aimed at verifying the effects of a short- and medium-term PEP for elementary school students on the theoretical knowledge, as well as the execution of ADL.

## METHOD

In this longitudinal study,^17^ the sample was defined by sample calculation, using the software G*Power and assuming moderate effect size (f=0.3), α=0.05, and power of 90%, which resulted in a minimum sample of 25 subjects. Considering the probability of loss in longitudinal studies, the initial sample consisted of 44 schoolchildren aged 8-12 years, mean age of 8.8±1.1 years, 54.5% (n=24) female, who attended the third grade of the elementary school of a state school in the city of Porto Alegre, Rio Grande do Sul. The selection of the school was by convenience and the research period was from April 2015 to November 2015.

In order to be included in the sample, students should be at least seven years old and be physically fit for the assessment of ADLs. The exclusion criterion was missing in one of the evaluative meetings in both assessment categories (video and test). All students voluntarily participated in the study, after receiving permission from parents or guardians, by signing the informed consent form. The study is part of a larger project named Posture Education Program for Schoolchildren, which was approved by the Ethics and Research Committee of the *Universidade Federal do Rio Grande do Sul* (UFRGS) (CAAE: 15356913.2.0000.5347) and seek to implement a PEP for schoolchildren in state schools of the State of Rio Grande do Sul.

Subjects were evaluated at three different moments:

- Before attending the PEP (pretest).
- After attending the PEP (post-test).
- Five months after attending the PEP, immediately after completion of four review classes (five months follow-up).

The PEP which the subjects attended, was based on the Back School of the Physical Education Department of UFRGS,^18^ and also in the School of Adapted posture proposed by Candotti et al.^14^ The PEP is structured in ten meetings, as follows: one for the administration of the pretest, eight for the delivery of theoretical and practical content, and one for the application of the post-test. Each class lasted 90 minutes and took place once a week. The contents delivered in class involved the study of the structures, curvatures, and functions of the spine, and ADLs, such as remaining standing and walking, carrying a backpack, picking up objects from the ground, sitting, chewing, and lying down. Each class had the following dynamics: review of the homework, brief review of the previously delivered content, presentation of new content, practice on the new content, feedback about the class, and explanation of homework.

**Figure 1:**
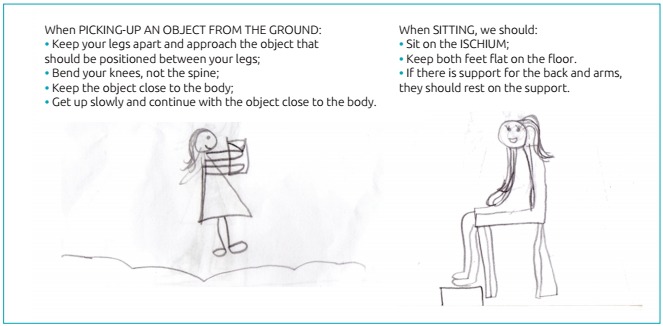
Drawings of a student who attended the Posture Education Program, in the leaflet developed in the follow-up.

The learning review was conducted three months after the end of the PEP and was structured in four meetings of 90 minutes each, with a 15-day interval. Theoretical and practical contents, which were developed during the PEP, were reviewed during those meetings. The same PEP teaching methodology was maintained for the review classes, except for the last meeting, during which a final review was carried out. In this final review, a brochure that illustrated the story of each class in relation to children’s learning about posture was used as the study material. This brochure was elaborated by the researchers according to topics generated by the students in the previous class (Figure 1). In the week after the fourth meeting, the students were re-evaluated according to the same post-test procedures, which characterized the five months follow-up.

Participants completed two types of examinations: a questionnaire to evaluate the theoretical knowledge of the spine and body posture, and a filmed circuit to evaluate the dynamic posture during ADL. In the pretest, only the assessment of the dynamic posture was performed. In the post-test and five months follow-up, all subjects of the sample completed both the assessments.

The questionnaire developed and administered by the researchers addressed questions concerning the structure of the spine and the ways to perform ADLs. It had 12 objective and descriptive questions, with a total score of 12 points, in which higher scores represent higher level of theoretical knowledge. Owing to the different topics included in the questions, they were divided into two sections: “anatomy” and “postural habits,” totaling 5 and 7 points, respectively. The first section included questions in which the participants should name regions of the spine and structures, such as ischium, vertebrae, and intervertebral disc, whereas in the second section, the participants needed to indicate appropriate and inappropriate attitudes represented in figures.

To verify the dynamic posture of the students during ADLs, the layout for assessing the dynamic posture - LADy was used. This is an instrument proposed and validated by Noll et al.^19^ The LADy enables the assessment of the dynamic body posture in schoolchildren in nine ADLs, by means of filming. In this study, we chose to evaluate only five ADLs:

1. Carrying the schoolbag.
2. Picking up an object on the ground.
3. Carrying the object.
4. Seated on a backless bench.
5. Seated in the chair to write.

**Figure 2:**
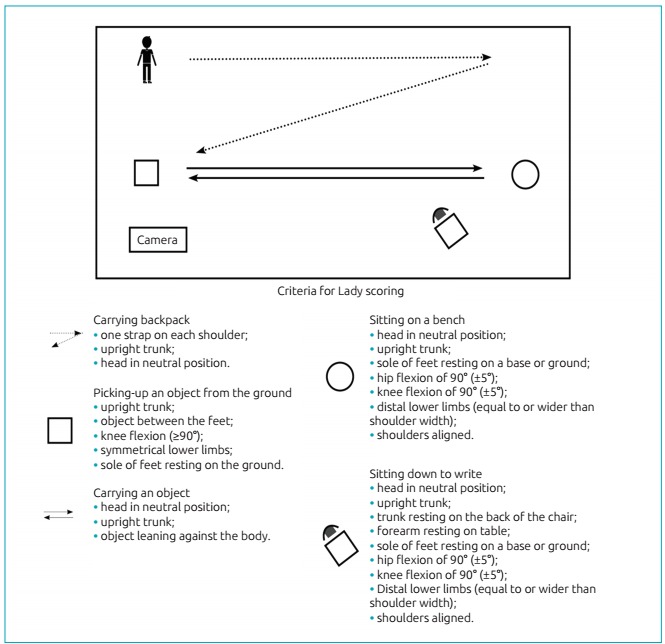
Scheme of the circuit layout for assessing the dynamic posture (LADy) to evaluate the dynamic posture, with description of the items scored in each activity of daily living. Each item observed scores 1 point in the score of each activity of daily living, according to the pre-established criterion in the LADy.

The participants were filmed while performing the LADy. The analysis of dynamic posture was performed later through the observation of the films, which was conducted by a single evaluator who was trained in the method. There are different elements which are given a score based on the analyzed posture. Considering the five ADLs evaluated in this study, each child could reach a maximum total score of 27 points (the higher the score achieved, the more adequate was the execution of ADL). The circuit used and the items evaluated in each ADL are shown in Figure 2.

Statistical analysis was performed using the software Statistical Package for Social Sciences (SPSS), version 20.0. Shapiro-Wilk test was applied to verify the normality of the data, in addition to descriptive statistics with mean and standard deviation (SD). The Friedman test (considering α<0.05) was applied to verify the differences between the evaluative moments; and in order to compare the different moments, post hoc Wilcoxon test and Bonferroni correction (α/3, where 3 is the number of comparisons performed - pretest *versus* post-test, pretest *versus* follow-up, and post-test *versus* follow-up*)* were performed*,* adopting α<0.017 at the post hoc, according to Field’s suggestion.^20^

## RESULTS

The initial sample consisted of 44 students of both sexes. Among them, 2 students did not respond to the questionnaire in one of the two times it was applied, 13 students failed to attend at least one evaluative moment of LADy, and 4 students did not participate in at least one stage of the two stages of the assessment. Therefore, considering the exclusion criteria, a final sample of 38 schoolchildren was obtained for evaluation by questionnaire, and another sample of 27 schoolchildren was obtained to assess the LADy.

In the analysis of the questionnaires, the Wilcoxon test showed no significant difference between the post-test and the follow-up when the sections of anatomy and postural habits were specifically compared, and in the overall score of the questionnaire (Table 1). Therefore, the students retained a similar level of theoretical knowledge about the spine in the questions related to anatomy or to postural habits in the final evaluation, and five months after PEP’s conclusion.

**Table 1:**
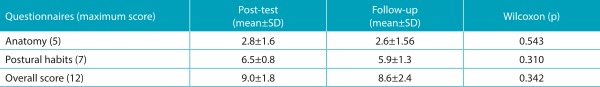
Mean scores and standard deviation obtained in the questionnaires, in the evaluation of anatomy and postural habits sections, and overall score in post-test and follow-up periods (n=38).

SD: standard deviation.

The results of the scores obtained in the evaluation of dynamic posture, in the pretest, post-test, and follow-up periods of each ADL studied and also the final score of the LADy are shown in Table 2. It can be observed that only the ADL carrying the schoolbag showed no significant difference among the evaluative moments.

**Table 2:**
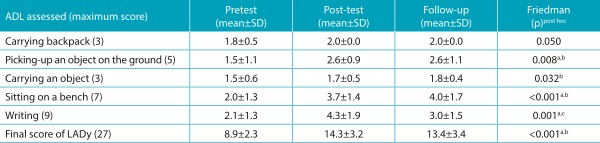
Mean scores and standard deviation obtained in the dynamic assessment for each activity of daily living, and the final score in the layout for assessing the dynamic posture (LADy), in pretest, post-test, and follow-up periods (n=27).

^a^Significant difference between pretest and post-test; ^b^Significant difference between pretest and follow-up; ^c^Significant difference between post-test and follow-up; SD: standard deviation; ADL: activities of daily living; LADy: layout for assessing the dynamic posture.

The *post hoc* Wilcoxon/Bonferroni showed significant difference between the scores of pretest and post-test for the variables picking up an object on the ground *(p*=0.008), writing *(p*<0.001), sitting on a bench *(p*<0.001), and for the final score of dynamic assessment *(p*<0.001), with no difference in the variable carrying an object *(p*=0.041). Significant difference was found between the pretest and follow-up periods for the variables picking up an object on the ground (*p*=0.008), sitting on a bench (*p*<0.001), carrying an object (*p*=0.005), and also for the final score of dynamic assessment *(p*<0.001), finding no significant difference in the position adopted during writing *(p*=0.019). When differences occurred, the differences showed better scores in post-test and follow-up periods, compared to the pretest scores.

## DISCUSSION

In the post-test and follow-up evaluations, participants achieved an average of 54% correct answers in the questions related to theoretical knowledge about the anatomy of the spine, which reveals the need to reinforce the learning about this content. However, with regard to the postural habits, the students performed better, scoring 88% correct answers, showing a good theoretical knowledge of postural habits. Moreover, there was no significant difference between the evaluative moments, suggesting that the theoretical knowledge acquired in the final evaluation concerning anatomical questions or postural habits was retained five months after the conclusion of PEP. Studies that assess the level of theoretical knowledge after the conclusion of a PEP are scarce;^16^ however, studies evaluating this item immediately after PEP’s conclusion have achieved satisfactory results.^14^ ^,^ ^15^ This was the case of a study conducted by Candotti et al.,^15^ which evaluated 28 adolescents who were divided into experimental and control groups. They observed that the experimental group improved their knowledge about the functioning of the spine and posture compared with the pre-experiment.

An important aspect that may have influenced the low scores obtained by students in the assessments, is the fact that this study was conducted in a public school, in which parents and/or children’s guardians did not have the habit of studying or helping their children with homework. However, Fonseca et al.,^21^ in a population-based study in a city in southern Brazil, found no significant association of the type of school (public or private) with the level of theoretical knowledge of posture; however, the discussion on the effect of posture education in different types of schools is still incipient, and it is important to conduct new studies.

By analyzing the scores obtained in the dynamic evaluation of each ADL, the ADL carrying backpack, regardless of the analyzed period, was the activity in which the students achieved a score closer to the maximum score (63% of the maximum on average), followed by the activity of carrying an object, whose average score was 56% of the maximum score. In the other postures (picking up an object on the ground, seated on a bench, and writing), the children achieved, on average, approximately 30% of the maximum score in the pretest period, and 50% of the maximum score on the post-test and follow-up periods. Therefore, it is important to emphasize that, despite the improvement of the results in the post-test and follow-up evaluations, the scores achieved by the children in the evaluation of dynamic posture are below the expectations. Therefore, it is evident that the review of PEP throughout school life of these children would be relevant as an attempt to increase these scores to levels closer to the maximum score.

Concerning the changes in the dynamic approach, this study found significant difference when comparing the final score of ADLs between PEP’s pretest and post-test, with improved results in the second evaluative moment, indicating an improvement in body posture during the performance of ADLs. This result is in line with those highlighted by a systematic review that included nine studies related to back schools developed for schoolchildren in Brazil. This systematic review found that those programs, in addition to contributing to the immediate improvement of theoretical knowledge of the subject, are also related to positive change in the dynamic posture of schoolchildren when assessed immediately after the intervention.^22^

Studies assessing the immediate effects of the PEP on theoretical knowledge, or performance of ADLs have been found more frequently in the literature, although with methodological differences with respect to the PEP structure or evaluation tools.^14^ ^,^ ^15^ On the other hand, studies that aim at evaluating the retention of these effects are still scarce. There is a study that aimed at evaluating the effects of a PEP for children and adolescents eight months after its completion. That study evaluated static posture, dynamic posture, and theoretical knowledge in the pre-intervention, post-intervention, and follow-up periods.^16^ With regard to the dynamic posture, the authors concluded that the knowledge acquired after the intervention was not retained in a period of eight months, demonstrating that an interval of this magnitude without guidance or learning reviews, probably affected negatively the program’s effects on the long term.^16^ In contrast, when we carried out the 5-months follow-up after PEP’s conclusion, we did not find a significant difference in the final score when comparing post-test and follow-up assessments, suggesting that improved dynamic posture found after PEP remained after five months. This maintenance of the results is possibly related to the learning review provided for the students.

In most cases, the performance of children with respect to the dynamic posture in the post-test and follow-up evaluations was higher when compared to the pretest, whereas in the comparison between post-test and follow-up, the performance was maintained. However, the posture of the students when carrying school supplies had a peculiar aspect. When assessing this posture, no significant difference was found in any comparison of the evaluative moments (Table 2). In other words, the posture adopted by the students when carrying their school supplies was not dependent of the study intervention. It is possible that this result is associated with the fact that the students already had adopted an adequate posture for carrying school supplies before attending the PEP. The ideal technique for the students to carry school supplies is to position the backpack at the height of the back, with the two straps tightly fitted over the shoulders.^23^

Ritter and Souza^24^ carried out a study that intended to verify how the students of the elementary schools in Porto Alegre carried school supplies and the weight carried by them. They found that the participants mainly used backpack supported on both shoulders, and that the average weight of school supplies carried was below the maximum limit suggested in the literature, which is 10% of body weight. In addition, the study of Silva Júnior et al.^25^ evaluated the same variables, but in a sample of students of the fifth year of a school of Petrolina, Pernambuco. They observed that 81% of students used backpack with two straps, and among those, 82.4% used both shoulders straps, showing a difference only in the results related to the weight carried. In that study, almost half of the evaluated students carried school supplies above the tolerable limit. These studies corroborate the idea that children are more aware of the proper execution of the activity of carrying school supplies, making it an ADL well assimilated by the students.

Considering that the improvement of dynamic posture found at the conclusion of the PEP was retained after five months, when a learning review was offered in the interval between the assessments, it is suggested that new interventions aimed at posture education also provide learning reviews. Basically, long intervals without reviewing the learning can lead to the gradual disuse of the adequate motor patterns, whereas learning reviews may favor the assimilation of the content.^16^

The main limitation of the study is the lack of evaluation of theoretical knowledge in the pretest. Considering that the research was carried out in the school environment, in the same classes shift, and embedded in the school routine, it was decided not to administer the questionnaire without previous delivery of the content, as this approach would not be in compliance with the school standards for tests application. Similarly, the lack of evaluation before carrying out the learning reviews after three months of PEP’s conclusion is another limitation.

In conclusion, the results of this study suggest that PEP has a positive effect when assessed immediately after its completion, when it was found that it improved the dynamic posture of the students for most of the assessed ADLs. In addition, the maintenance of these positive effects also occurred in the period of 5-months follow-up, after the implementation of learning reviews. With regard to the assessment of theoretical knowledge related to the spine and postural habits, the level of knowledge was retained between the final evaluation and the 5-months follow-up.
